# Pyrosequencing to Identify Homogeneous Phenomenon When Using Recipients/Donors with Different CYP3A5*3 Genotypes in Living Donor Liver Transplantation

**DOI:** 10.1371/journal.pone.0071314

**Published:** 2013-08-08

**Authors:** King-Wah Chiu, Toshiaki Nakano, Kuang-Den Chen, Chia-Yun Lai, Li-Wen Hsu, Ho-Ching Chiu, Ching-Yin Huang, Yu-Fan Cheng, Shigeru Goto, Chao-Long Chen

**Affiliations:** Liver Transplant Program, Kaohsiung Chang Gung Memorial Hospital, and Chang Gung University, College of Medicine, Taiwan, Republic of China; The University of Hong Kong, Hong Kong

## Abstract

This study used pyrosequencing to determine the proportional distribution of CYP3A5*3 genotypes to further confirm the homogeneous phenomenon that is observed when recipients and donors in living donor liver transplantation (LDLT) have a different single nucleotide polymorphism (SNP) genotype. We enrolled 42 recipient/living donor pairs and the SNPs of CYP3A5*3 were identified by polymerase chain reaction-restriction fragment length polymorphism. We performed 120 liver graft biopsies as part of clinical investigations after LDLT. Pyrosequencing of the CYP3A5*3 SNPs revealed that among the 16 recipients with the G/G genotype, 94.68% had the G and 5.32% the A allele. Among the 14 recipients with the A/G genotype, 78.08% had the G and 21.92% the A allele, and among the 12 recipients with the A/A genotype, 18.45% had the G and 81.55% the A allele. Among the 12 donors with the G/G genotype, 93.85% had the G and 6.14% the A allele. Among the 26 donors with the A/G genotype, 75.73% had the G and 24.27% the A allele, and among the 4 donors with the A/A genotype, 11.09% had the G and 88.91% the A allele. There were a total of 120 liver graft biopsy samples; among the 37 recipients with the G/G genotype, 89.74% had the G and 10.26% the A allele, among the 70 recipients with the A/G genotype, 71.57% had the G and 28.43% the A allele, and among the 13 recipients with the A/A genotype, 48.25% had the G and 51.75% the A allele. The proportional distribution of G and A alleles of the CYP3A5*3 SNP between recipients/donors and liver grafts after LDLT was significantly different (p<0.001). Pyrosequencing was useful in identifying detailed proportional changes of the CYP3A5*3 SNP allele distribution, and to confirm the homogeneous phenomenon when recipients and donors in LDLT have a different genotype.

## Introduction

We previously reported that inter-individual differences in the activity and expression of the metabolizing enzyme cytochrome P450 (CYP) *2C19* considerably contributed to clinical drug pharmacokinetics in the setting of living donor liver transplantation (LDLT) [1]–[2]. However, Western blot and polymerase chain reaction (PCR) with restriction fragment length polymorphism (RFLP) do not produce satisfactory results of quantitative changes in the different genotypes between recipients and the liver grafts [3]. The homogeneous phenomenon is an interesting biological genetic change found in situations such as after LDLT [4]. *CYP3A5* has been demonstrated to be closely associated with tacrolimus concentrations [5]–[6]. Variability in the activity of the haplotypes of the CYP3A5*3 gene has been reported to result from genetic polymorphisms encoding their genes [7]–[8]. Herein, we present the results of a prospective pyrosequencing study conducted to clarify the proportional changes of the CYP3A5*3 haplotypes in the A/A, A/G, and G/G genotypes to further confirm the homogeneous phenomenon in the setting of LDLT.

## Methods

We performed 120 liver biopsies in 42 recipients because of evidence of abnormal liver function after LDLT, as previously reported [2], [9]. Among the 42 recipients (27 males and 15 females), 10 were children and 32 were adults with a mean age of 42.62 years (range, 3–69 years). The serum levels of tacrolimus (ng/mL) and cyclosporine A (ng/mL) and complete liver function including measurements of alanine transferase, aspartate transferase, total bilirubin, prothrombin time with international normal range, and albumin were assessed on postoperative day 1 (POD1) and postoperative day 30 (POD30) after surgery. According to the haplotypes of the SNPs from PCR-RFLP analysis, the positions of the CYP3A5 polymorphisms were explored at rs776746. Sixteen recipients had the G/G, 14 had the A/G, and 12 had the A/A genotype. Among the 42 living donors, 12 had the G/G, 26 had the A/G, and 4 had the A/A genotype. Among the 120 liver biopsies from liver grafts after LDLT, 37 had the G/G, 70 had the A/G, and 13 had the A/A genotype (Table 1). All of the patients received a donor graft from a recipient with a different genotype. Recipients with the same genotype were excluded from this study.

**Table 1:** Liver function and serum drug levels of tacrolimus/cyclosporine A from postoperative day 1 to postoperative day 30 after living donor liver transplantation.

Category	Recipient (n = 42)		*P* value
Age (mean) (range)	42.62 (3–69)		
Sex M:F	27∶15		
	*D1*	*D30*	
ALT	252.35±446.40	82.40±244.43	<0.001
AST	292.42±660.86	65.54±300.20	<0.001
T-Bil	7.19±11.71	1.24±3.60	<0.001
PT (INR)	1.91±3.34	1.05±0.40	<0.001
Alb	3.06±0.88	3.74±0.95	<0.001
Tacrolimus (ng/mL) (n = 32)	2.51±2.73	6.17±9.58	<0.001
cyA (ng/mL) (n = 10)	283.89±308.93	1058.30±582.37	<0.001

D: donor; R: recipient; D1: post liver transplantation day 1; D30: post liver transplantation day 30.

### Genomic DNA Isolation

Genomic DNA was isolated from 0.5 mL of EDTA-treated whole blood and liver biopsy samples using a QIAamp DNA mini kit (Qiagen) in accordance with the manufacturer’s instruction. The repository of the DNA dataset was the NIH Short Read Archive, and the reference SNP (refSNP) was rs776746 (http://www.ncbi.nlm.nih.gov/SNP/snp_ref.cgi?rs=776746).

### PCR-RFLP for CYP3A5*3 Genotyping

A PCR assay using forward primer (5′-CATGACTTAGTAGACAGATGAC-3′) and reverse primer (5′-GGTCCAAACAGGGAAGAAATA-3′) was performed in a 25 µL of reaction volume. The PCR conditions consisted of initial denaturation at 94°C for 7 min, followed by 35 cycles of denaturation at 94°C for 30 s, annealing at 55°C for 30 s, extension at 72°C for 30 s, and a final extension step at 72°C for 7 min. The obtained PCR product had a length of 293 bp and was digested for 3 h at 37°C using the restriction enzyme Ssp. The digested products were separated on 4% agarose gels. Samples with the CYP3A5 *1/*1 genotype gave bands of 148, 125, and 20 bp in length, whereas samples with the *CYP3A5* *3/*3 genotype gave bands of 168 and 125 bp in length.

### Pyrosequencing for CYP3A5*3 Genotyping

#### 1. DNA amplification

One of the primers used for amplification of DNA for PCR analysis was biotinylated. Primers for pyrosequencing were designed with PyroMark Assay Design Software 2.0. In the PCR assay (PyroMark PCR Kit-Qiagen), we used a forward primer (5′-TGTACCACCCAGCTTAACGA-3′) and a reverse primer that was biotinylated at the 5′ end (5′-GGTCCAAACAGGGAAGAAAT-3′). The assay was performed in a 25-µL reaction volume. The PCR conditions consisted of initial denaturation at 95°C for 15 min, followed by 45 cycles of denaturation at 94°C for 30 s, annealing at 60°C for 30 s, extension at 72°C for 30 s, and a final extension step at 72°C for 10 min. The PCR products were separated on 2% agarose gels. The obtained product had a length of 98 bp.

#### 2. Pyrosequencing analysis

**Figure 1:**
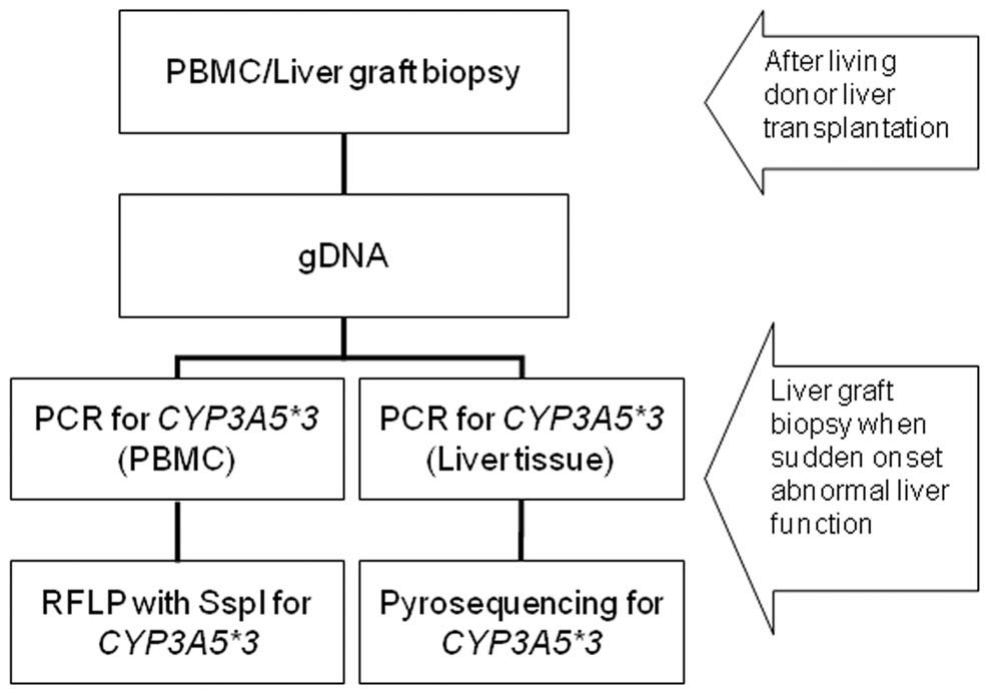
The single nucleotide polymorphism of CYP3A5*3 was identified by polymerase chain reaction-restriction fragment length polymorphism analysis. Pyrosequencing was performed with peripheral blood mononuclear cells and liver tissues of graft biopsies.

Biotinylated PCR products were immobilized on streptavidin-coated Sepharose beads (Streptavidin Sepharose High Performance, GE Healthcare). All of the streptavidin-coated Sepharose beads (2 µL per sample) were mixed with binding buffer (40 µL per sample) in a tube. High-purity water was then added to a total volume of 80 µL per well, including the PCR product (20 µL). This immobilization mix was incubated for 10 min at 25°C with continuous mixing (1,400 rpm) on a shaking device, and the sequencing primer was then diluted to 0.3 µM in annealing buffer. Next, 25 µL of the solution was transferred to each well of a PyroMark Q24 Plate. After immobilization, the liquid was removed by aspirating the beads with a Vacuum Prep Tool and the beads were treated for approximately 5 s with 75% ethanol, 5 s with denaturation buffer, and 5 s with washing buffer. The PyroMark Q24 Plate containing the samples was heated at 80°C for 2 min using a PyroMark Q24 Plate Holder and a heating block. The plate was then removed from the plate holder and the samples were allowed to cool to room temperature (15–25°C) for at least 5 min, and the reagents, including enzyme and substrate mixtures, and nucleotides were added to the cartridge (PyroMark Q24,Qiagen) [10]. The samples were analyzed using a PyroMark Q24 system (Qiagen) according to standard protocols. The order of nucleotide dispensation was chosen based on suggestions provided by the PyroMark Assay Design Software 2.0 (Figure 1).

### Ethics Statement

This research was conducted in accordance with the Declaration of Helsinki (2000) of World Medical Association and institutional standards and was granted ethical approval by the institute review board from Chang Gung Memorial hospital (No: 98-4051B). Written informed consent for participation in the study was obtained from participants or from a parent or guardian in case of minor participants. All of the participants had been provided and obtained the written informed consent to participate in this study and the ethics committees had approved all of the consent procedure.

### Statistical Analysis

Statistical analyses were performed using SPSS software (version 12.0; SPSS, Chicago, IL, USA). Comparisons of parameters of the haplotypes of CYP3A5*3 between the donors and recipients were performed using the *X* ^2^ test, Fisher’s exact test, and Student’s *t*-test. *P* values less than 0.05 were considered statistically significant.

## Results

We performed 120 liver graft biopsies in the 42 recipients, 15 of which were performed within 1 month (12.5%) and 105 after more than 1 month after transplant (87.5%). They were performed once in 15 recipients, twice in 7, 3 times in 9, 4 times in 1, 5 times in 6, 6 times in 1, 7 times in 2, and 10 times in 1. The results of pyrosequencing of the 42 recipients were 94.68±4.26% with the G and 5.32±4.26% with the A allele among the 16 recipients with the G/G genotype, 78.08±5.34% with the G and 21.92±5.34% with the A allele among the 14 recipients with the A/G genotype, and 18.45±17.16% with the G and 81.55±17.16% with the A allele among the 12 recipients with the A/A genotype. Among the 42 living donors, 93.85±3.59% had the G and 6.14±3.59% had the A allele among the 12 donors with the G/G genotype, 75.73±7.62% had the G and 24.27±7.62% had the A allele among the 26 donors with the A/G genotype, and 11.09±8.25% had the G and 88.91±8.25% had the A allele among the 4 donors with the A/A genotype. There was no statistically significant difference in the distribution of genetic polymorphisms between the recipients and living donors. In the 120 liver graft biopsies, 89.74±5.64% had the G and 10.26±5.64% had the A allele among the 37 samples with the G/G genotype, 71.57±13.42% had the G and 28.43±13.42% had the A allele among the 70 samples with the A/G genotype, and 48.25±13.46% had the G and 5.75±13.46% had the A allele among the 13 samples with the A/A genotype. There were significant changes in the distribution of G and A alleles in the G/G and A/A genotypes between the recipients and liver graft biopsy samples (p<0.001 and p<0.001) and the donors and liver graft biopsy samples (p = 0.011 and p<0.001). In the A/G group, the p value was 0.039 between the recipients and liver graft biopsy samples, and 0.237 between the donors and liver graft biopsy samples (Table 2).

**Table 2:** Results of single nucleotide polymorphism analysis of the frequency of G and A alleles of the recipients, donors, and liver graft biopsy samples after living donor liver transplantation.

	Recipient			Donor			Liver graft biopsy			P value	
SNP	No	G (%)	A (%)	No	G (%)	A (%)	No	G (%)	A (%)	R:Lg	D:Lg
G/G	16	94.68±4.26	5.32±4.26	12	93.85±3.59	6.14±3.59	37	89.74±5.64	10.26±5.64	<0.001	0.011
A/G	14	78.08±5.34	21.92±5.34	26	75.73±7.62	24.27±7.62	70	71.57±13.42	28.43±13.42	0.039	0.237
A/A	12	18.45±17.16	81.55±17.16	4	11.09±8.25	88.91±8.25	13	48.25±13.46	51.75±13.46	<0.001	<0.001
Total	42			42			120				

Abbreviations: SNP: single nucleotide polymorphism; R: recipient; D: donor; Lg: liver graft.

**Figure 2:**
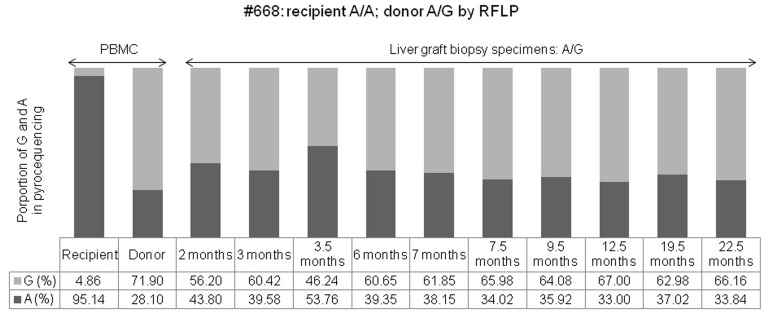
The proportional changes of G and A alleles were determined by pyrosequencing of peripheral blood mononuclear cells of a recipient (#668) and a donor, and of a liver graft biopsy sample after living donor liver transplantation (recipient: A/A; donor: A/G, identified by restriction fragment length polymorphism analysis).

For transplantation number 668, the donor CYP3A5*3 genotype was A/G and the proportional distribution by pyrosequencing was 28.1% for the A and 71.9% for the G allele, while the genotype of the recipient was A/A and the distribution was 95.14% for the A and 4.86% for the G allele. We performed 10 liver biopsies at 2, 3, 3.5, 6, 7, 7.5, 9.5, 12.5, 19.5, and 22.5 months after LDLT because of abnormal liver function. A significant proportional change of the A/G genotype of CYP3C5*3 was found by pyrosequencing. which showed a proportional increase in A to 43.8, 39.58, 53.76, 39.35, 38.15, 34.02, 35.92, 33.0, 37.02, and 33.84% from an initial 28.1%, and a proportional decrease in G to 56.2, 60.42, 46.24, 60.65, 61.85, 65.98, 64.08, 67, 62.98, and 66.16% from the initial 71.9% following liver graft biopsy, respectively. Although the A/G genotype was the same as that of the donor, the proportional distribution in the biopsy samples was significantly different in the pyrosequencing analysis when compared with that of the donor (Figure 2).

**Figure 3:**
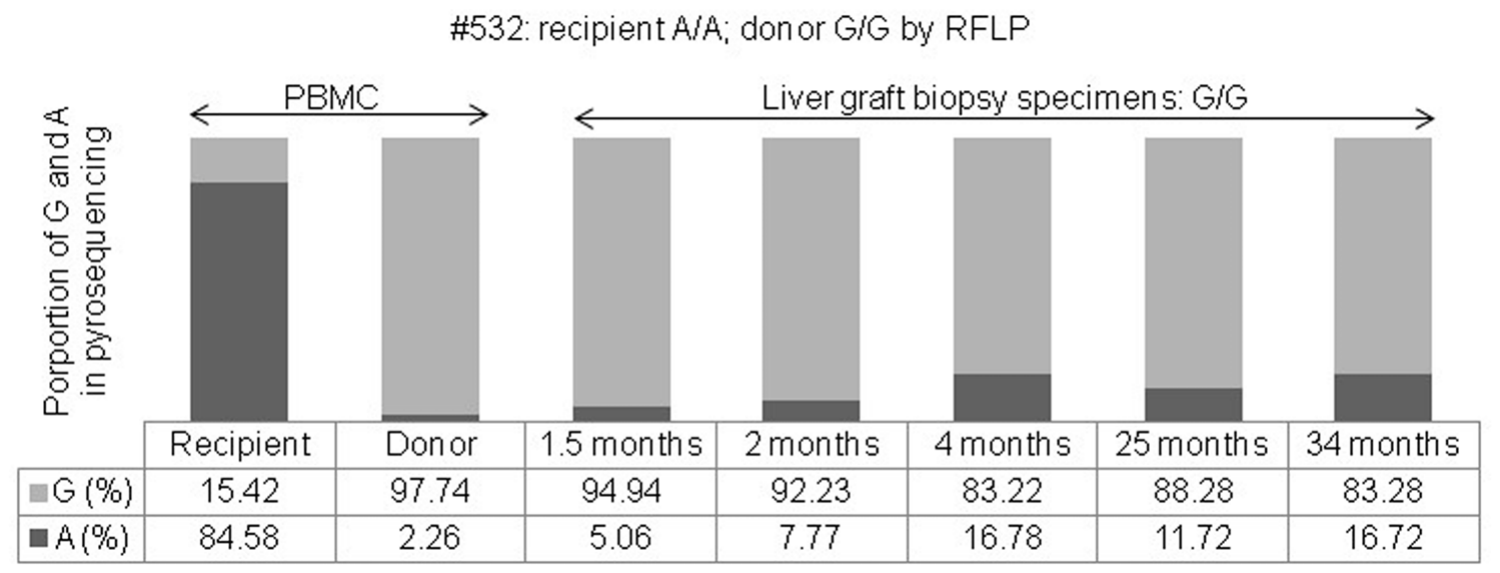
The proportional changes of G and A alleles were determined by pyrosequencing of peripheral blood mononuclear cells of a recipient (#532) and a donor, and of a liver graft biopsy sample after living donor liver transplantation (recipient: A/A; donor: G/G, identified by restriction fragment length polymorphism analysis).

For transplantation number 532, the donor CYP3C5*3 genotype was G/G and the proportional distribution by pyrosequencing was 2.26% for the A and 97.74% for the G allele, while the genotype of the recipient was A/A and the distribution was 84.58% for the A and 15.42% for the G allele. Five liver biopsies were performed at 1.5, 2, 4, 25, and 34 months after LDLT because of abnormal liver function. A significant proportional change of the G/G genotype of CYP3C5*3 was found by pyrosequencing, which showed a proportional increase in A to 5.06, 7.77, 16.78, 11.72, and 16.72% from the initial 2.26%, and a proportional decrease in G to 94.94, 92.23, 83.22, 88.28, and 83.28% from the initial 97.74% following liver graft biopsy, respectively. Although the G/G genotype was the same as that of the donor, the proportional distribution in the biopsy samples was significantly different in the pyrosequencing analysis when compared with that of the donor (Figure 3).

**Figure 4:**
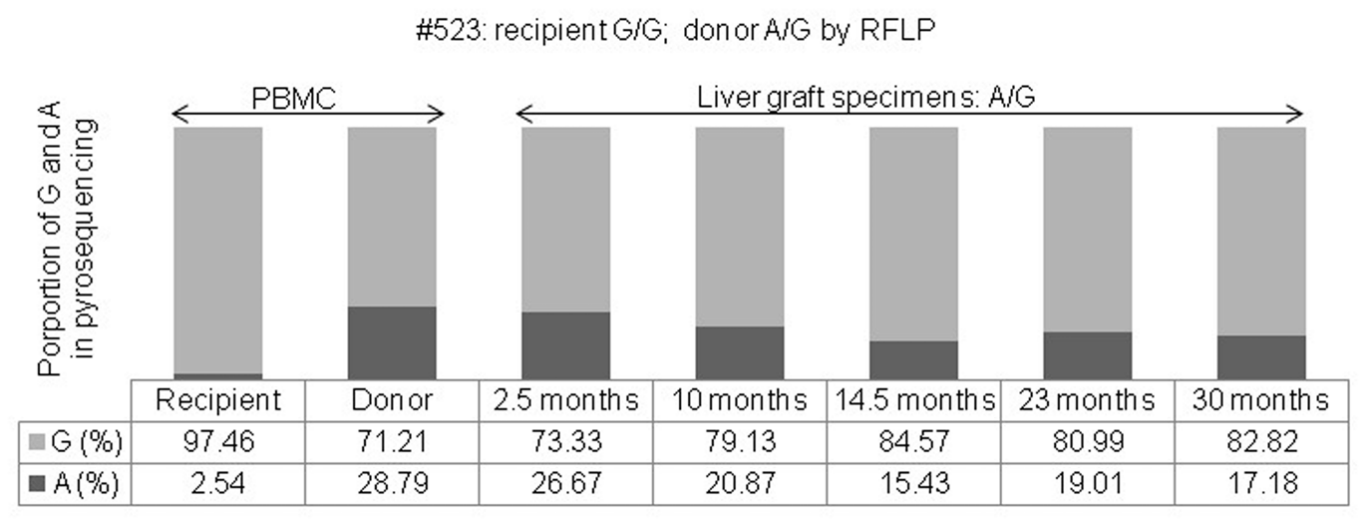
The proportional changes of G and A alleles were determined by pyrosequencing of peripheral blood mononuclear cells of a recipient (#523), a donor, and a liver graft biopsy sample after living donor liver transplantation (recipient: G/G; donor: A/G, identified by restriction fragment length polymorphism analysis).

For transplantation number 523, the donor CYP3C5*3 genotype was A/G and the proportional distribution by pyrosequencing was 28.79% for the A and 71.21% for the G allele, while the genotype of the recipient was G/G and the distribution was 2.54% for the A and 97.46% for the G allele. Five liver biopsies were performed at 2.5, 10, 14.5, 23, and 30 months after LDLT because of abnormal liver function. A significant proportional change of the A/G genotype of CYP3C5*3 was found by pyrosequencing, which showed a proportional decrease in A to 26.67, 20.87, 15.43, 19.01, and 17.18% from the initial 28.79%, and a proportional increase in G to 73.33, 79.13, 84.57, 80.99, and 82.82% from the initial 71.21% following liver graft biopsy, respectively. Although the A/G genotype was the same as that of the donor, the proportional distribution in the biopsy samples was significantly different in the pyrosequencing analysis when compared with that of the donor (Figure 4).

## Discussion

Compared with RFLP, pyrosequencing has the advantages of high precision and accuracy [10], and can clarify in detail the proportional changes in the G and A alleles of SNPs. In this study, the proportional distribution of the G allele of the CYP3A5*3 G/G genotype was more than 90%, and that of the A allele less than 10% in the recipients as well as in the donors before LDLT. After LDLT, the proportional distribution of the G allele was less than 90% and that of the A allele more than 10% in the G/G genotype of CYP3A5*3 in liver graft tissues (Table 2). Basically, the original CYP3A5*3 genotype of the liver graft did not change as it depended on the donor, however the proportional changes of the distribution of the G and A alleles were significantly modified by the different genotypes (A/G or A/A) of the recipients. This is the so-called homogeneous phenomenon which we previously reported in our study on *CYP2C19* [4]. We previously investigated changes in the different genotypes of *CYP2C19* with Western blot and directed sequencing methods, however the results were inconclusive [3]. In the current prospective study, we focused on the characterizations of the genetic polymorphisms of CYP3A5*3 using RFLP, followed by pyrosequencing, which can clarify the proportional changes in the distribution of the G and A alleles in detail when the recipients and donors in LDLT have a different SNP genotype. However, we did not perform hepatocyte isolation. It is the true that the microdissection of hepatocytes can identify the unique genotypes from liver grafts. However, in a real world setting, liver graft tissues mix with the recipient’s peripheral blood creating a natural evolution, the so-called homogeneous phenomenon, which is the focus of the present study. Where the isolation of graft hepatocytes should belong to the donor, it may not represent the real situation after LDLT.

Although the serum levels of tacrolimus and cyclosporine A were lower on POD1 after liver transplantation, the serum levels of both were increased on POD30. The homogeneous phenomenon of drug metabolic isoenzymes may play a major role in providing physiological regulation to avoid acute rejection [2], [4], [9]. In a recent bone marrow stem cell study, direct DNA sequencing confirmed that phenotype changes were caused by mutations [11]. In our previous study [4], all recipients underwent sequential graft liver biopsies, and DNA sequencing analysis showed that all of the final liver *CYP2C19* genotypes of the recipients depended on those of the donors, but were not related to the original characteristic genotype of the recipients after LDLT. Direct sequencing with amplitude changes in the G and A wave did not accurately represent the power of the evidence of change. For transplantation number 532 in this study, the recipient had the CYP3A5*3 genotype A/A (84.58% of A and 15.42% of G, by pyrosequencing) received a liver graft from a donor with the G/G genotype (2.26% of A and 97.74% of G, by pyrosequencing) in LDLT. After transplantation, the liver graft still showed the G/G genotype in RFLP analysis, however, a proportional increase in A and a decrease in G was found by subsequent pyrosequencing analysis at 1.5 months and a marked proportional change at 4 months after LDLT. This phenomenon was also found in transplantation number 523, where a recipient with the G/G genotype received a liver graft from a donor with the A/G genotype, as well as in transplantation number 668, where a recipient with the A/A genotype received a liver graft from a donor with the A/G genotype of CYP3A5*3. Regarding the timing of liver graft biopsy, most cases (105/120, 87.5%) were performed more than 1 month after LDLT, which should be sufficient to demonstrate replacement of the drug metabolic system P450 by the new graft with stationary homogeneous phenomenon due to different genotypes of the recipients and donors. Therefore, high serum levels of tacrolimus/cyclosporine A and a more normalized liver function were found on POD30. It has been reported that the status of the *CYP2C19* genotype can affect the efficacy of proton pump inhibitors in the treatment of peptic diseases [12], [13] and when used in combination with tacrolimus in transplantations [14], [15]. In our recent study, we reported that the unstable function of CYP3A4*18, CYP3A5*3, and *MDR1-3435* of the new liver graft in the initial stage of LDLT may be maintained by interaction of the new liver graft and the intestinal origin [16], [17]. In this situation, drug metabolic function may not be completely stable resulting in lower serum levels of tacrolimus/cyclosporine A and high liver function test results. After more than 1 month, a functional new liver graft presents with significantly higher serum levels of tacrolimus/cyclosporine A resulting in normal liver function as well as completed homogeneous phenomenon. Accordingly, once the proportional changes of the distributions of G and A alleles are constant, subsequent pyrosequencing studies of the recipient represent the dosage and serum levels of tacrolimus/cyclosporine A that are sufficient to avoid acute rejection after LDLT [18], [19]. The homogeneous phenomenon is difficult to measure, especially its association with intra-variation variability at an early stage after LDLT. Longitudinal follow-up biopsies showing that the percentage SNP genotypes has reached a stable distribution, especially after POD30, should overcome this intra-variation variability. The novelty of the findings in the current study is important for the recipients receiving anti-rejection agents, in whom the metabolism of the given drug dosage should be taken into consideration. The clinical benefits and consequences of measuring differences in genotypes provided the rationale behind this study, as well as the implications for whether the recipients should receive higher/lower doses of anti-rejection agents to ensure a functional graft after LDLT.

In conclusion, pyrosequencing of liver grafts after LDLT can provide information to clarify the proportional changes of the distribution of the A and G alleles of the CYP3A5*3 SNP when the SNP genotype of the recipients/donors is different. Pyrosequencing and RFLP analysis are distinct methods providing different information. Further studies are required to confirm that the CYP3A5*3 homogeneous phenomenon truly occurs when recipients and donors have different SNP genotypes.
